# A69 LONG-TERM EFFECTS OF TORKELSON SYNDROME

**DOI:** 10.1093/jcag/gwab049.068

**Published:** 2022-02-21

**Authors:** S E Dinn, I Wrobel, S Bhandal, M Brundler

**Affiliations:** Pediatric Gastroenterology, University of Calgary, Calgary, AB, Canada

## Abstract

**Background:**

Torkelson Syndrome is a rare, familial enteropathy with villous edema causing recurrent episodes of life threatening vomiting and secretory diarrhea in childhood, with children being well between episodes. The only description, of a Mennonite family from southern Alberta, suggests normal growth and no long term sequelae. Severe laboratory abnormalities common during bouts of diarrhea (hypoalbuminemia, neutropenia and electrolyte abnormalities) normalize in the asymptomatic phase except for decreased IgG subclasses. Typical findings from the duodenum and jejunum do not vary between acute episodes and when well. The pathophysiology is poorly understood, with acute episodes thought to be triggered by a non-specific infection in the setting of IgG2 deficiency.

Apolipoprotein A-IV (APOA4) mutation has been documented in Torkelson syndrome. APOA4 is exclusively expressed in proximal small intestine villous enterocytes. It is thought to be involved in regulating pathways that control inflammation in enterocytes, resulting in decreased inflammation. It may prevent recruitment of leukocytes to inflamed intestinal enterocytes by suppressing P-selectin in the endothelial cells. Fully functioning APOA4 decreases secretion of IL-4 and TNF-a. In theory, abnormalities in APOA4, could result in a robust, unopposed inflammatory response in the small intestine.

**Aims:**

Report possible long-term sequelae of Torkelson Syndrome.

**Methods:**

Case report.

**Results:**

13 year old male with Torkelson syndrome presents with short stature and iron deficiency anemia responsive to oral iron. His weight is at the 0.1%ile, height at the 0.4%ile and BMI at the 1.6%ile.

Life time hypoalbuminemia (20g/L) indicates chronic protein-losing enteropathy. Vitamin D is chronically low but responds to oral supplementation. IgA and IgM are normal, but IgG and its subclasses are decreased. Liver enzymes and electrolytes, including calcium and phosphate, outside of episodes of diarrhea are normal.

Endoscopy shows prominent, thick villi, with fern-like appearance in the duodenum with normal stomach, esophagus, terminal ileum and colon. Biopsy results are consistent with previously described features of Torkelson syndrome with no additional abnormalities. MRE shows mild dilatation of the jejunum with hypertrophic conniventes with fat content. Abdominal ultrasound shows hepatomegaly with coarse echotexture and normal elastography. He has delayed bone age and low bone density.

**Conclusions:**

Patients with Torkelson syndrome, despite their recurrent episodes of life threatening vomiting and secretory diarrhea in childhood, were thought to have no long term sequelae including normal growth.

Our patient, presents with chronic iron deficiency anemia, short stature, delayed bone age and density, and hepatomegaly of unknown significance. This case may broaden our knowledge of this poorly described condition to inform long term management of these patients.

**Funding Agencies:**

None

